# Ultrafast optical integration and pattern classification for neuromorphic photonics based on spiking VCSEL neurons

**DOI:** 10.1038/s41598-020-62945-5

**Published:** 2020-04-08

**Authors:** Joshua Robertson, Matěj Hejda, Julián Bueno, Antonio Hurtado

**Affiliations:** Institute of Photonics, University of Strathclyde, 99 George St, Glasgow, G11RD United Kingdom

**Keywords:** Optics and photonics, Semiconductor lasers

## Abstract

In today’s data-driven world, the ability to process large data volumes is crucial. Key tasks, such as pattern recognition and image classification, are well suited for artificial neural networks (ANNs) inspired by the brain. Neuromorphic computing approaches aimed towards physical realizations of ANNs have been traditionally supported by micro-electronic platforms, but recently, photonic techniques for neuronal emulation have emerged given their unique properties (e.g. ultrafast operation, large bandwidths, low cross-talk). Yet, hardware-friendly systems of photonic spiking neurons able to perform processing tasks at high speeds and with continuous operation remain elusive. This work provides a first experimental report of Vertical-Cavity Surface-Emitting Laser-based spiking neurons demonstrating different functional processing tasks, including coincidence detection and pattern recognition, at ultrafast rates. Furthermore, our approach relies on simple hardware implementations using off-the-shelf components. These results therefore hold exciting prospects for novel, compact and high-speed neuromorphic photonic platforms for future computing and Artificial Intelligence systems.

## Introduction

Neuromorphic computing has seen a surge in interest for data intense processing tasks for which brain-inspired artificial neural networks (ANNs) have proven very powerful^1^. Demand for Artificial Intelligence (AI) and machine learning systems, using ANNs for operation, has dramatically exploded with increasingly challenging applications (e.g. AI assistants, autonomous vehicles, big data, meteorological predictions and assistive robotics)^2–4^. However, traditional Von Neumann computing architectures struggle to achieve the efficiency and parallelism required to recreate complex ANNs^5^. Hence, neuromorphic computing platforms made up of artificial spiking neurons and synapses, supported by mature electronic technologies, have attracted increasing interest. Current systems, such as TrueNorth^6^, SpiNNaker^7^, Neurogrid^8^ and HICANN^9^, have each demonstrated impressive performance. However, neuromorphic electronic realisations suffer from the same limitations as modern day microprocessors: slowed progress of Moore’s law^10^ and use of electrical signals (which inherently limits bandwidth, speed, communication distance and energy efficiency)^11^.

Consequently, neuromorphic photonic approaches have started to emerge given their unique advantages, e.g. ultrafast performance, large bandwidths, low cross talk, and high parallelism. Light enabled operation of brain-inspired photonic systems means that speeds up to 9 order of magnitude faster than biological neurons, and crucially up to 6 orders of magnitude faster than electronic approaches^12^, can be achieved. Due to the appealing properties of these photonic systems, growing interest has given rise to many reports of electro-optic artificial synaptic devices^13,14^ and spiking photonic neuronal models based on numerous different systems, e.g. phase change materials^15^, resonant tunnelling diodes^16,17^ and different types of semiconductor lasers^18–27^. However, to date, demonstrations of functional systems, based on spiking photonic neurons with continuous operation, remain elusive.

This work provides a first report of a leaky integrate and fire (LIF) spiking photonic neuron based upon a Vertical-Cavity Surface-Emitting Laser (VCSEL-neuron) performing functional tasks (e.g. coincidence detection and pattern recognition) at ultrafast sub-ns rates (using ~100 ps input signals) with continuous operation. Furthermore, our approach is based on VCSELs, ubiquitous devices in today’s consumer electronics products. VCSELs are used in mobile phones for auto-focus and facial recognition functionalities, in supermarket barcode scanners, etc. Hence, there is great potential for new developments that could add intelligent processing to existing VCSEL-based systems. Moreover, our approach uses hardware friendly implementations relying solely on off-the-shelf components at telecom wavelengths; hence making it fully compatible with optical communication and data centre technologies. Thus, this work opens new research paths towards future photonic ANN hardware architectures based on VCSEL-neurons for ultrafast AI and neuromorphic computing platforms.

## Results

### Artificial Spiking VCSEL-neuron

**Figure 1:**
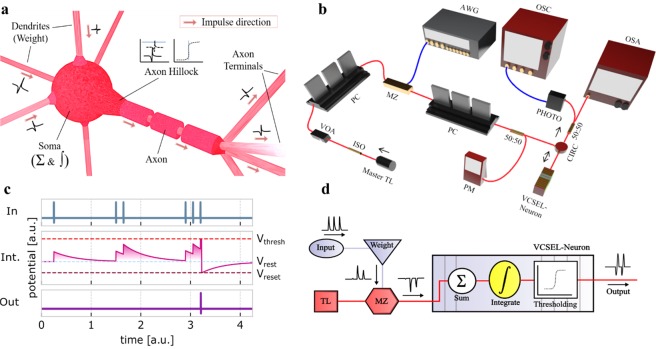
Spiking VCSEL-neuron. (**a**) Illustration of a biological neuron. (**b**) Experimental setup used to investigate a spiking VCSEL-neuron under external optical injection of intensity-encoded stimuli. Optical fibre connections are shown in red and electrical connections in blue. (**c**) Idealistic depiction of the LIF model. Inputs injected into the device (In) are integrated (Int.), with a time constant decay, towards a threshold potential (red dotted line). When the threshold requirement is met, the system fires a spiking response (Out) and the potential reaches the reset value (dark red) before returning to its resting potential (light blue). (**d**) Flow diagram of the VCSEL-neuron. Optical injection is encoded with pre-weighted inputs. These are integrated over time in the VCSEL-neuron where a spike activation function thresholds inputs before firing.

Figure 1a depicts the concept behind the proposed LIF spiking VCSEL-neuron. Neurons in the LIF model are subject to multiple synaptic inputs, where each of the inputs (dendrites) is individually weighted. The weighted inputs are later summed and temporally integrated in the soma (cell body) to deliver a specific spiking output. The integrated inputs contribute to the build-up of a membrane potential within the neuron that is governed by an activation function. In the LIF model, the summed inputs decay over time (leaky) due to what is called membrane potential decay. The firing of the neuron is only achieved when the threshold potential is exceeded by the integrated inputs. After the firing of a spike, the neuron potential drops (to the reset potential) before returning to the resting potential. The spiking output is communicated via the neuron’s axon. Diagrams of a biological neuron and the operation of the LIF model are shown in Fig. 1a,c. A LIF photonic spiking neuron is thus required to perform weighting, summation, temporal integration, thresholding and spiking.

Figure 1b,d plot respectively the experimental setup used to investigate spiking LIF VCSEL-neurons and their operation principle. Input light signals are temporally-encoded with stimuli, in the form of ~100 ps pulses at Full Width Half Maximum (FWHM). A customisable numerical array allows for the weight-control of the input stimuli. These are summated and integrated by the VCSEL-neuron which responds by firing a ~100 ps long spike upon exceeding the threshold energy for spike activation. Full details on the experimental setup, the VCSEL’s properties and their operation as spiking neurons are provided in the Methods section and the Supplementary Information.

### Multiple input integration

**Figure 2:**
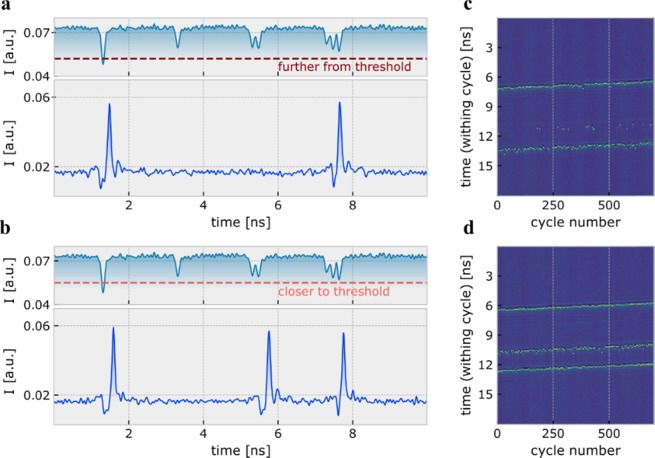
Integration of multiple pulses with varying amplitude. Optically injected input (top) and the recorded optical response from the VCSEL-neuron (bottom) when using (**a**) far from threshold and, (**b**) close to threshold operation points. The input injection contains first a large and a small control pulse before a doublet a triplet pulse input burst. (**c**,**d**) Temporal maps that plot the continuous time traces of a and b as cycling patterns. Spike intensity is represented using colour, with yellow and blue colours showing respectively spike crests and stable output intensities. The maps provide an illustration of the statistical repeatability of the VCSEL-neuron’s response.

As is true in biological neurons, the ability to integrate multiple inputs is essential for neural network functionality. Here, we show that a spiking VCSEL-neuron delivers this key ability at ultrafast rates. We demonstrate that a VCSEL-neuron is able to integrate bursts of 100 ps input pulses and fire short spikes depending on the amplitude of the incoming signals (see Fig. 2). Specifically, the top and bottom plots in Fig. 2a,b show respectively the measured time series of the encoded optical input and the corresponding optical output of the VCSEL-neuron, where each plot is configured with a different spike activation threshold level. Both Fig. 2a,b show initially a stable response until the arrival of a large-amplitude control pulse (at ~1.5 ns). This exceeds the activation threshold of the VCSEL-neuron, eliciting a spike at its output (see supplementary information). A second small-amplitude control pulse (at ~3.5 ns) represents a subthreshold input which does not trigger a spike. After the two control pulses, a doublet and a triplet pulse burst enter the VCSEL-neuron. For the higher spiking threshold case, Fig. 2a shows the activation of two spikes at the VCSEL-neuron’s output. The first spike is triggered by the large control pulse at the beginning of the input waveform. The second spike is triggered by the successful integration of the individual sub-threshold pulses in the triplet burst, whose combined energy exceeds the system’s spiking threshold. For the case shown in Fig. 2a, the combined energy of the two sub-threshold inputs in the doublet burst is lower than the spiking threshold and therefore does not trigger a spike. The temporal map of Fig. 2c shows the response of the VCSEL-neuron when the input pattern in Fig. 2a enters the device 750 consecutive times. Yellow dots on the map represent spike crests with (yellow) lines illustrating consistent responses. From the map, we can see consistent spiking responses from the control pulse and the triplet burst, however, the doublet pulse burst does not elicit spiking responses regularly across all input cycles. The time series and temporal map of Fig. 2b,d, on the other hand, demonstrate that when the spike firing threshold is lower, and therefore a reduced total integrated energy is required to cross the activation threshold, the doublet burst can also reliably trigger spiking responses. Similarly, the consistency of the spiking outputs generated by the triplet burst increases, with all inputs triggering a spiking event. Figure 2 demonstrates the energy integration and thresholding functionalities of the LIF VCSEL-neuron and the tunability of its response, by controlling either the amplitude of the incoming stimuli or the threshold for spike activation.

### Temporal-coincidence detection

**Figure 3:**
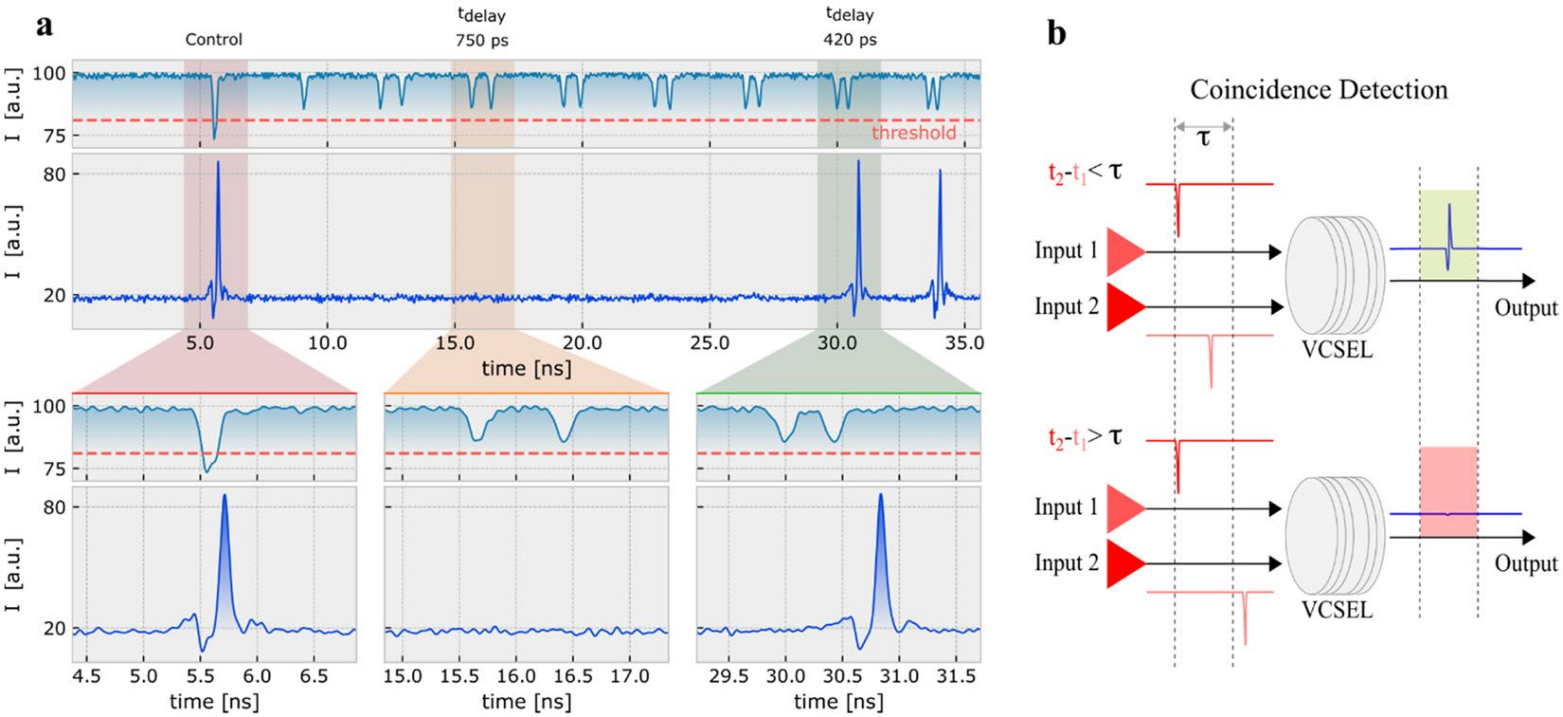
Coincidence detection of time delayed inputs in the VCSEL-neuron. (**a**) Input encoded optical injection (top), recorded time trace response from the VCSEL-neuron (middle) and corresponding expanded insets (bottom). The encoded input includes large and small control pulses and 7 pairs of subthreshold input pulse pairs. The separation between input pulse pairs (t_delay_) decreases from ~820 ps to ~340 ps. The threshold level is indicated approximately by the dotted red line. (**b**) Coincidence detection concept diagram. t_1_ and t_2_ are the corresponding arrival times of inputs 1 and 2, and τ represents the maximum integration period that successfully activates a spiking response from the system.

We explore now the effect of temporally uncorrelated inputs arriving into the VCSEL-neuron. Figure 3a shows the optical input pattern used in this experiment (top plot). This input is comprised of pulse pairs with decreasing delay time (t_delay_) from ~820 ps to ~340 ps. Figure 3a also plots the spiking response from the VCSEL-neuron (middle plot) with expanded insets at the bottom. These show that a first spike (bottom-left inset in Fig. 3a) is triggered by the arrival of an initial super-threshold control pulse. The second input pulse pair with t_delay_ ~750 ps fails to trigger a spike as its combined energy is not sufficient to exceed the spike activation threshold (bottom-middle inset of Fig. 3a). In contrast, the sixth input pulse pair elicits a fast spiking response from the VCSEL-neuron (bottom-right inset in Fig. 3a). Similarly, the seventh input pulse pair (t_delay_ ~340 ps) also triggers a spike firing event. These results indicate that for large t_delay_ values, the system fails to sufficiently integrate sub-threshold input pulse pairs beyond the spiking threshold. Temporal maps depicting the system’s response to multiple consecutive input patterns are provided in the Supplementary Information. These show that the smaller the delay time (t_delay_) between pulses, the more consistent the achieved spiking responses. This points to the VCSEL-neuron working as a leaky system where the resultant integrated amplitude degrades over time. The maximum temporal integration period is dictated by the requirement that the total integrated energy must be greater than the sum of the threshold potential and the inherent leaky loss. Input pulses that satisfy this condition will always trigger a spike, and we expect larger spiking thresholds to result in the requirement of larger input pulses or shorter integration periods.

Programmable coincidence detection is an important use of a selective integration period. Coincidence detection, used in neurons to help combat timing jitter^28^, is the detection of two temporally close sub-threshold inputs that only activate an output when arrival times fall within a short temporal window. Figure 3b depicts schematically the concept of coincidence detection applied to our VCSEL-neuron: when the arrival time difference between the second and first pulses (t_2_ - t_1_) is less than the integration time (τ), the system triggers an output, or remains silent otherwise. The VCSEL-neuron in Fig. 3a therefore performs coincidence detection, where two inputs, detected within ~420 ps, fire a spike. Alarm triggering is one of the most common applications of coincidence detection and by using the VCSEL-neuron, it would be possible to generate 100 ps long spiking responses to multiple temporally-encoded input alerts. Similarly, this functionality can be used to create optical spiking logic gates, which can be incorporated into larger network configurations towards decision making circuits similar to those traditionally implemented in electronics, but at much higher speeds.

### Ultrafast pattern recognition

**Figure 4:**
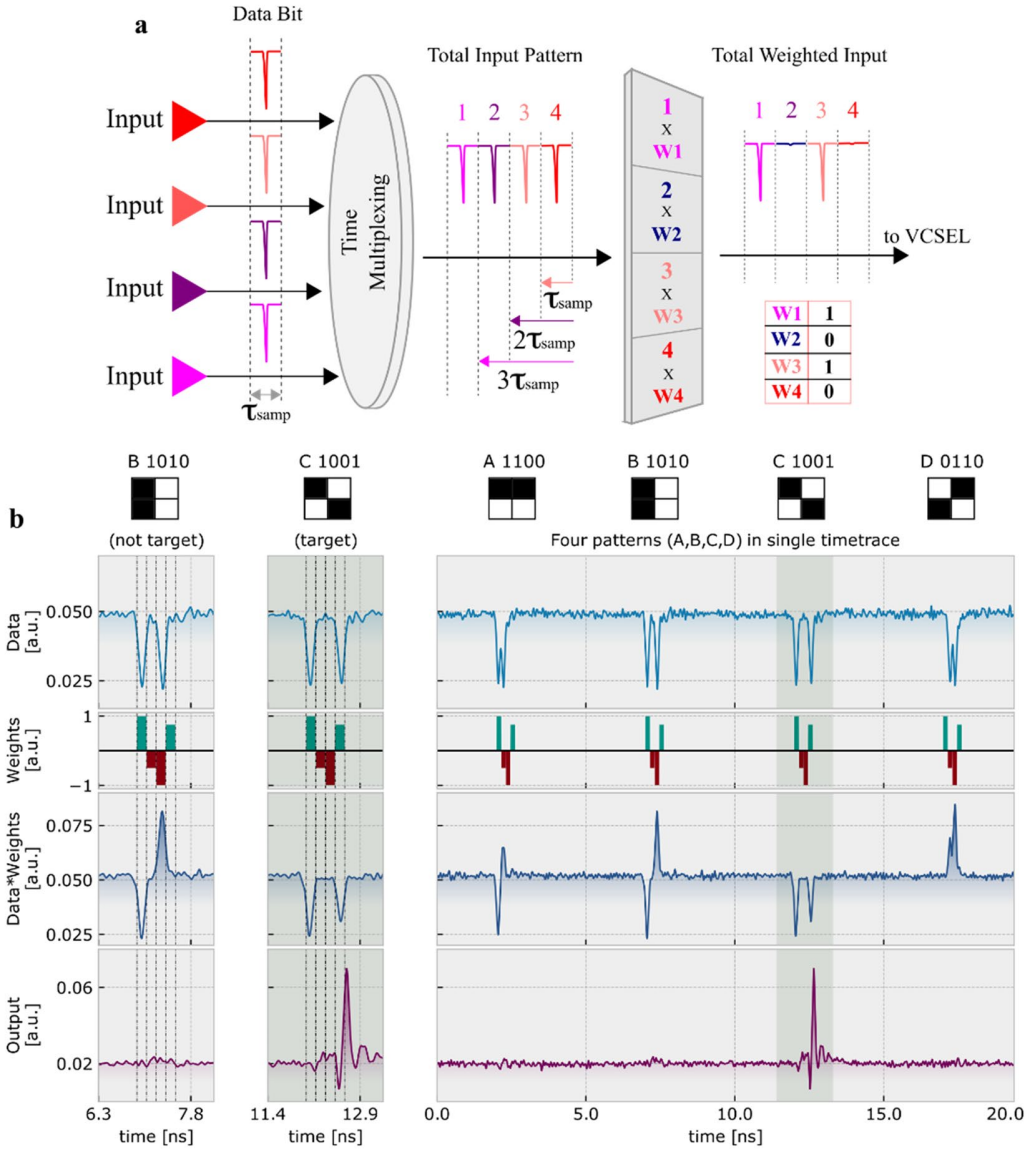
Pattern Recognition with a spiking VCSEL-neuron. (**a**) Diagram demonstrating the time division multiplexing (TDM) and weighting concepts used to encode 4-bit input data patterns before injection into the VCSEL-neuron. Four virtual inputs are sampled (τ_samp_) and staggered into a single input line, creating 4-bit data patterns which are weighted using a customisable array. After weighting, the input data patterns are injected into the VCSEL-neuron for processing. (**b**) Experimentally recorded time series showing the pre-weighting optical inputs (Data), the selected weighting values (Weights), the post-weighting optical inputs (Data * Weights) and the optical output of the VCSEL-neuron (Output). Four pattern combinations are shown with two active ‘1’ bits, namely A (1100), B (1010), C (1001) and D (0110), with pattern C (1001) the target for recognition.

**Figure 5:**
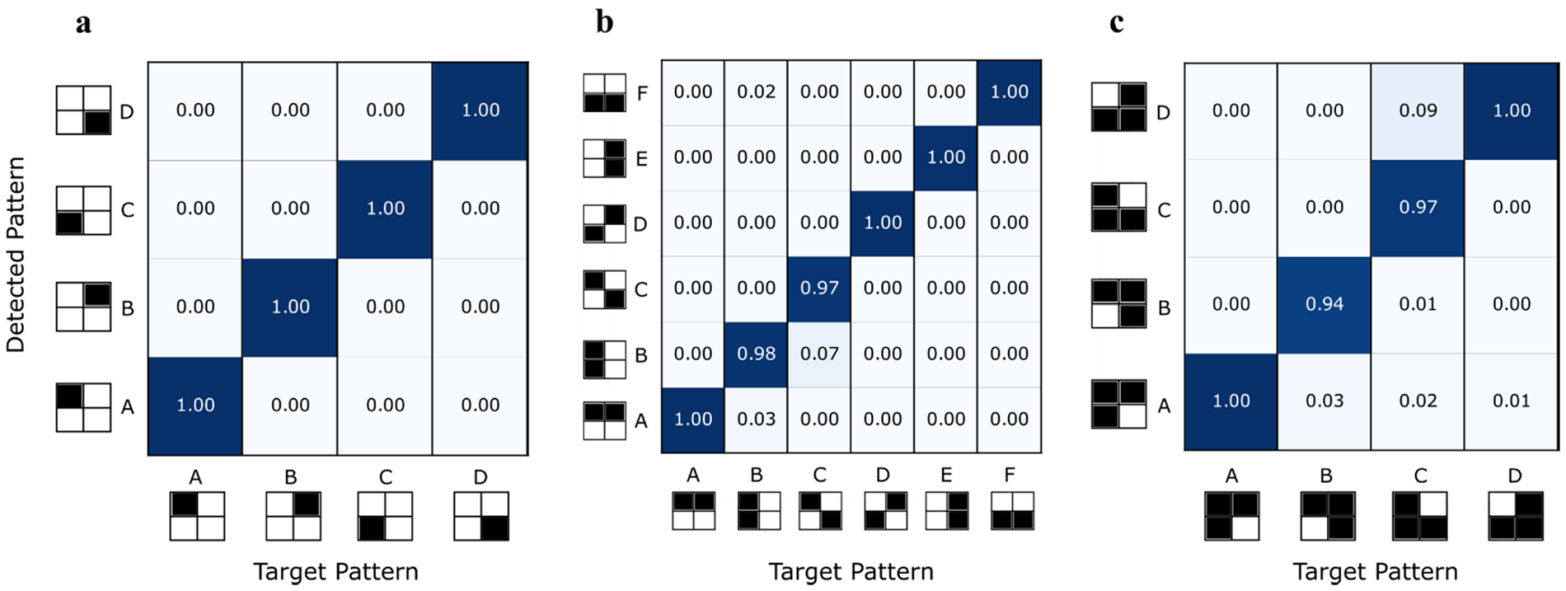
Pattern Recognition Confusion Matrices. Measured detection/recognition efficiencies for all 4-bit data patterns investigated. (**a**) The input data set contained 4 patterns with 1 active ‘1’ bit, (**b**) 6 patterns with 2 active ‘1’ bits and **c** 4 patterns of 3 active ‘1’ bits. The (blue) colour depth indicates the recognition efficiency over 130 consecutive test cycles.

Neuromorphic photonic architectures have the potential to transform information processing and AI systems by providing an ultrafast platform that could improve the speed of intense computational tasks such as speech, image and pattern recognition. We focus on this key issue, performing a pattern recognition task at GHz rates with a single continuously operated (non-gated) VCSEL-neuron. Specifically, Figs. 4 and 5 demonstrate the recognition of target 4-bit data patterns. Figure 4a illustrates schematically the technique implemented for this task, which uses time division multiplexing (TDM) to encode a sequence of four ~100 ps pulses, each representing a ‘1’ or a ‘0’ bit, arriving from four different (virtual) inputs. The 4-bit patterns are weighted prior to their injection into the VCSEL-neuron, which will only fire a spike in response to the target pattern, remaining silent for other non-target patterns. In this work we focus on the recognition of individual target patterns and apply unique sets of weights to incoming data. In this work we also group patterns such that recognition can be achieved in one classification step. For full details on the pattern generation, TDM and weighting steps, see the Methods section and the Supplementary Information.

Figure 4b plots the recognition of a specific pattern with two active ‘1’ bits, pattern C (1001). The time series in the top row of Fig. 4b shows four input data patterns with two active ‘1’ bits, namely A (1100), B (1010), C (1001) and D (0110), before weighting; the second row shows the weight values, which for this particular case were equal to 1, −0.5, −1 and 0.75; and the third row plots the data patterns after weighting. The weighted patterns are optically injected into the VCSEL-neuron, which produces the response shown in the bottom row in Fig. 4b. This shows that a single spike is fired following the arrival of target pattern C (1001), with none of the other three patterns eliciting a response.

We have tested the recognition efficiency of the system for 14 different 4-bit pattern combinations. For each of them, we investigated the VCSEL-neuron’s response to 130 consecutive recognition tasks, and compared the expected and detected spiking responses. The recognition efficiencies for all input patterns are provided in the confusion matrices in Fig. 5a–c, which reveal both successful and false recognitions. We must note that false recognitions appear as non-zero efficiencies for non-matched target and detected patterns. Specifically, Fig. 5a shows the recognition efficiencies of all input patterns with one active ‘1’ bit. For these cases, the weighting step eliminates the ‘1’ bits of all non-target patterns, leaving only the target pattern with a single input pulse. Therefore, for this case, no false recognitions occur as shown in Fig. 5a.

Figure 5b plots the confusion matrix of all patterns with two active ‘1’ bits, showing very high recognition efficiencies across all 130 tests. Figure 5b shows that the best performance is achieved for patterns A (1100), D (0110) and F (0011), which are characterised for having two consecutive ‘1’ bits. This means the VCSEL-neuron integrates the energy of these active bits across the minimum possible integration time, reducing the effects of the leaky system. The overall contribution to the activation function is therefore stronger creating more efficient spiking responses. Very good performance is also obtained for patterns B (1010), C (1001) and E (0101), but with slightly higher number of false recognitions, given that the larger separation time between active ‘1’ bits requires a longer integration time. In Fig. 5c the recognition efficiencies for the three active ‘1’ bit patterns are provided. Similar to the results in Fig. 5b, patterns A (1110) and D (0111) show good consistency and low false recognitions, as the consecutive bits help to minimise the effect of the leaky system. The performance of both other patterns, B (1101) and C (1011), shows again very good target recognition, but with marginally higher false recognitions.

In this work, a separation between bits of ~150 ps (corresponding to pulse widths of 100 ps FWHM) was used as it was the maximum available bandwidth provided by our experimental equipment. This permitted the recognition of 4-bit patterns within the sub-nanosecond temporal integration window of our VCSEL-neuron (see Fig. 3). However, we must note that in principle, our system could also be used to detect patterns with higher numbers of bits by improving integration capabilities and input data rates. Reducing input pulse width and input sampling period would allow more pulses to be integrated in a shorter period, reducing the effect of the leaky system. Additionally, to better illustrate the fast performance of our system, a small sub-nanosecond (~830 ps) time separation between consecutive input data patterns was configured (see Supplementary Information). The results in Fig. 5 demonstrate the functional application of our proposed VCSEL-neurons in a pattern recognition task at GHz rates based upon the system’s integration capabilities. We believe these results, together with the high prospects for integration of VCSEL-neurons into increasingly complex architectures, in both off-chip (fibre-based) and on-chip (array-based) realisations, offer great prospects for ultrafast brain-inspired pattern recognition and computing platforms for AI functionalities.

## Discussion

We report experimentally on a spiking VCSEL-neuron capable of integrating multiple time-delayed inputs; hence operating similar to LIF neuronal models. We also demonstrate a functional neuromorphic photonic processing system with a hardware-friendly implementation based upon a single VCSEL-neuron with operation at sub-nanosecond speeds. We first demonstrate that a VCSEL-neuron can perform coincidence detection for ultrafast alarm triggering and decision making systems. We then demonstrate a 4-bit pattern recognition task at GHz rates (using 100 ps pulses to encode input bits) with continuous operation and with very high recognition efficiencies. Motivated by the presented results, we hope to investigate the scaling of our current system towards multi-VCSEL-neuron operation, for the running of parallel recognition and classification tasks, as well as the potential for neuromorphic dynamics on-chip at the micro-scale. Furthermore, the benefits of VCSELs, e.g. low fabrication costs, compactness, ease of integration into off and on-chip arrays, operation at telecommunication wavelengths, etc., are complimentary to the ultrafast speed of the neuronal spiking dynamics they can yield (multiple orders of magnitude faster than the timescales of biological neurons). Hence, there is significant potential for new developments where intelligent processing may be added to ubiquitous VCSEL-based technologies already found in many everyday applications, e.g. mobile phones for auto-focus and facial recognition functionalities, supermarket barcode scanners, data centres for internet communications, etc. The true potential of these devices is becoming increasingly apparent and with these promising functionalities, sights are set on spiking VCSEL-neuron systems for future ultrafast photonic information processing, machine learning and AI platforms.

## Methods

### Experimental setup

The LIF VCSEL-neuron in this work is investigated using the experimental setup included in Fig. 1b. A tunable laser (Santec TLS-210V) is used to generate constant optical signals at a desired wavelength in the 1300 nm infrared window. The tuneable laser emission is passed through an isolator (ISO), to avoid undesired reflections, and through a variable optical attenuator (VOA) and polarisation controllers (PC), to respectively control the input power and polarisation of the optical signal. This is encoded with programmatically-weighted intensity stimuli using a Mach-Zehnder amplitude modulator and a 12 GSa/s arbitrary waveform generator (Keysight M8190a). The optical line is divided into two paths via a 50:50 fibre directional coupler. The first one is connected to a power meter (PM) to monitor the injected intensity, and the second one is connected to an optical circulator (CIRC) which injects the stimuli-encoded optical signals into the VCSEL-neuron. The circulator also collects the VCSEL-neuron’s output for post-synaptic analysis with a 9.5 GHz amplified photodetector (Thorlabs PDA8GS), a real-time 13 GHz oscilloscope (Agilent Infiniium DSO81304B) and an optical spectrum analyser (Anritsu MS9710C). A commercially-available VCSEL with emission wavelength at 1300 nm was used as the VCSEL-neuron. Throughout this work the VCSEL-neuron was driven with 5.0 mA bias current and kept at a constant temperature of 298 K. Characterisation measurements of the VCSEL’s emission are provided in the Supplementary Material.

### Multiple input integration

To investigate the integration of multiple inputs, the VCSEL-neuron was injected with an input waveform comprised of four different input signal combinations (separated each by 2 ns). These consisted first of a large- and a small-amplitude control pulses, and bursts of two and three small-amplitude input pulses. Pulses within the doublet and triplet bursts were configured with a peak to peak separation of ~160 ps with burst lengths equal to ~400 ps and ~600 ps, respectively. The input waveform was encoded into the injection of the VCSEL-neuron as described previously. For this analysis, results were measured for two different spike firing threshold levels. These different spiking threshold levels were set by altering the frequency detuning between the optically injected signal and the VCSEL’s resonance. An injection power of 183 μW into the orthogonal mode of the VCSEL was used for both the far from threshold (−6.0 GHz) and close to threshold (−6.35 GHz) measurements.

### Coincidence detection

For the temporal coincidence analysis, the input waveform used was generated with a set of input control pulses, followed by two identical sub-threshold input pulses (~170 ps, FWHM), whose delay time (t_delay_) was decreased from ~820 ps to ~340 ps. Similarly, this pattern was generated using the AWG and encoded in the optical input using the Mach-Zehnder modulator, prior to its injection into the VCSEL-neuron. The experiment was conducted at an optical injection power of 244.2 μW and a frequency detuning of −9.18 GHz from the orthogonal mode of the VCSEL.

### Ultrafast pattern recognition

Time division multiplexing (TDM) was used to encode sequences of four ~100 ps pulses, producing 4-bit patterns with a total length of ~650 ps. In our scheme, input ‘1’ and ‘0’ bits correspond to high- and zero-amplitude negative pulses, respectively. The 4-bit patterns were split into three input data sets, where every combination of 1, 2 and 3 active ‘1’ bits were tested against one another in a single input waveform. The 4-bit patterns were weighted in a supervised scheme. User-controlled weights, that held a positive or negative value between 1 and −1, were multiplied with each of the 4-bits in the input data patterns, changing their intensity (see Fig. 4a for an example of applying a ‘1010’ weight array). Data sequences, comprised of 130 consecutive cycles, repeated the desired combination of 1, 2 or 3 active ‘1’ bit patterns for investigation. The output of the AWG was injected into the Mach-Zehnder modulator using the setup in Fig. 1b, where the weighted patterns were encoded into the optical input for their injection into the VCSEL-neuron. In this experiment an injection power of 140.5 μW was used at a frequency detuning of −7.41 GHz from the orthogonal mode of the VCSEL.

## Supplementary information

Supplementary information.

## Data Availability

The datasets generated and/or analysed during this study are openly available from the University of Strathclyde KnowledgeBase: 10.15129/919e2583-3da3-44a2-a444-fd0423b5d267.
