# Supplementary material for: Ultrafast optical integration and pattern classification for neuromorphic photonics based on spiking VCSEL neurons
Source: Sci Rep. 2020 Apr 8;10:6098. doi: 10.1038/s41598-020-62945-5 (PMC7142074; doi:10.1038/s41598-020-62945-5)
Supplement: Supplementary file 1 — Supplementary information. [file 41598_2020_62945_MOESM1_ESM.docx]

**Ultrafast optical integration and pattern classification for neuromorphic photonics based on spiking VCSEL neurons**

*J. Robertson^1^, M. Hejda^1^, J. Bueno^1^ & A. Hurtado^1^*

Institute of Photonics, University of Strathclyde, 99 George St., Glasgow, United Kingdom, G11RD

Supplementary Information

**Table of Contents**

1. **Vertical-Cavity Surface Emitting Lasers (VCSELs)**
2. **Characterisation Setup**
3. **Spiking Activation Threshold in VCSEL-Neurons**
4. **Coincidence Detection - Temporal Maps**
5. **Generation and Weighting of Input Data Patterns**
6. **Confusion Matrices**
7. **4-bit Pattern Representation**

**1. Vertical Cavity Surface Emitting Lasers (VCSELs)**

In the presented manuscript a single mode vertical-cavity surface-emitting laser (VCSEL) device was used. This device, sourced from RayCan ltd. (SN: RC230341-FFP-12321306), was tested and characterised prior to the operation demonstrated in the manuscript. Fig. S1 plots the lasing spectra and the VCSEL’s output power measured at different bias currents and at room temperature (293 K). Fig. S1a plots the L-I curve of the device measured at 293 K, showing a threshold current of I_th_ = 2.96 mA. When biased above threshold the device delivers continuous wave (CW) light emission, yielding for example 104 μW of optical output power when operated at 5.0 mA. The slope efficiency of the device was measured to be 51.2. Fig. S1b shows the red shift of lasing spectra with increasing applied bias current. The VCSEL used in this work was a single-longitudinal and single-transverse mode laser source, which had two coexisting orthogonally-polarised modes, referred here as the orthogonal (λ_x_) and parallel (λ_y_) modes. The device operates with a dominant λ_y_ mode and does not exhibit bias-induced polarisation switching across the measured operating parameters. At 5.0 mA the peak wavelength of the dominant λ_y_ mode measured 1303.15 nm and the peak of the subsidiary λ_x_ mode measured 1303.39 nm. The corresponding wavelength separation between the two orthogonally-polarised modes for this devices was measured and found equal to 0.24 nm (42.4 GHz).


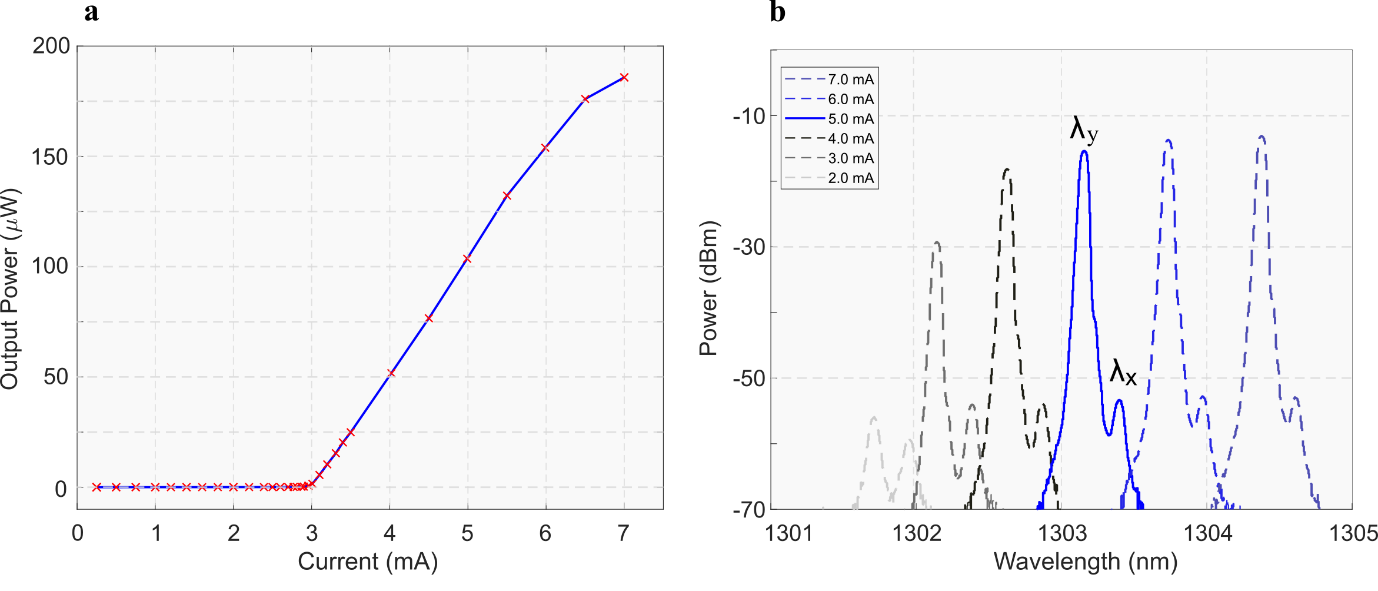


**Fig. S1 | VCSEL characterisation.** **a** L-I curve of the 1300nm-VCSEL used in this work, plotting the output power as a function of applied bias current. **b** Lasing spectra of the VCSEL for multiple bias currents. λ_x_ (λ_y_) denotes the orthogonal (parallel) polarisation mode of the device. All results were taken at room temperature (293 K).

**2. Characterisation Setup**

The experimental setup, shown in Fig. 1b of the main article file is made up entirely of commercially-available fibre-optic components. Optical injection was provided by a tuneable laser (TL) (Santec TLS-210V) and was fed through, an optical isolator (ISO), a variable optical attenuator (VOA) and a polarisation controller (PC). Pre-weighted inputs were generated using a 12 GSa/s arbitrary waveform generator (AWG) (Keysight M8190a) and encoded within the optical injection using a 10 GHz Mach Zehnder intensity modulator (MZ) (Thorlabs, LN82S-FC). An optical circulator (CIRC) was included to redirect the external optical injected signals into the VCSEL-neuron as well as to collect its output light. This was sent to analysis where it was converted to an electrical signal using a 9 GHz amplified photodetector (PHOTO) (Throlabs PDA8GS), and measured using a 13 GHz real time oscilloscope (OSC) (Agilent Infiniium DSO81304B). A power meter (PM) and an optical spectrum analyser (OSA) (Anritsu MS9710C) were used to provide measurements of injection power and detuning frequency. The total injection power was controlled using the VOA, and PCs were used to align of the polarisation of optical injection to that of the target VCSEL mode.

In this work, information is encoded within optical injection in the form of intensity variations. To achieve the encoding of information an arbitrary waveform generator and a Mach Zehnder intensity modulator were used. The arbitrary waveform generator (AWG) allowed for the generation of custom electrical signals down to a sample separation of 83.3 ps at 12 GSa/s. Therefore when generating the shortest pulses possible for injection into the VCSEL-neuron, a total of 3 samples were used in a ‘trough-peak-trough’ arrangement. This 3 sample combination permitted us to generate output pulses with a FWHM of ~100 ps. The AWG utilized amplified channels to produce output pulse amplitudes of ~750 mV when using half the DAC range (0 to 1), and ~ 1.5 V when using the full DAC range (-1 to 1). In our experiments half the DAC range (0 to 1) was utilized when generating positive electrical pulses (spike-activating drops in the optically injected signal), and the other half (DAC 0 to -1) when generating negative electrical pulses (spike-inhibiting steps in the optically-injected signal). The generated electrical signals were further amplified using a 10 dBm amplifier (Mini-circuits, ZX60-14012L-S+, 300 KHz-14 GHz), prior to being encoded into the intensity of the optical injection by means of the Mach Zehnder modulator. The latter was used to encode positive electrical pulses as intensity drops and negative electrical pulses as intensity steps in the externally injected optical signals entering the VCSEL-neuron.

**3. Spiking Activation Threshold in VCSEL-Neurons**

One of the key behaviours observed in biological neurons is the ability to trigger spiking signals in response to incoming stimuli exceeding a particular intensity threshold. Similar to biological neurons, our VCSEL-neuron exhibits this behaviour (but at ultrafast sub-nanosecond speeds), requiring incoming signals (or stimuli) with large-enough inputs to cross an activation threshold before a spiking output can be fired. We show this feature here by exposing our VCSEL-neuron to seven consecutive optical input pulses, each of increasing intensity. This experiment allows us to clearly observe and determine the spike activation threshold in our system.

In Fig. S2a we show the encoded optical injection and the corresponding response from the VCSEL-neuron. Each pulse in the optical injection was generated using 3 sample points and had a corresponding FWHM of ~100 ps. The intensity (strength) of the pulses increases from 0.4 to 1 times half the DAC range (0 to 1) of the AWG. Optical injection was made into the orthogonal (λ_x_) mode of the VCSEL-neuron with a detuning of -6.71 GHz and a total injection power of 164.8 μW, similar to the experimental conditions in the manuscript.

In response to the optically-injected signal shown in Fig. S2a (top), the VCSEL-neuron fires 4 spikes (Fig. S2a (bottom)). The fast spiking responses are generated at the output of the VCSEL-neuron for input strengths greater than 0.7. The spike efficiency curve, that calculates the percentage of spikes measured against the total number of input pulses, is plotted as a function of input pulse strength. From this graph, created using 653 sets of input pulses, we can clearly see the formation of an activation function. Similar to the results plotted in Fig. S2b, input strengths greater than 0.7 produce consistent spiking responses, indicating these pulses have exceeded the spike firing threshold of the device. These results verify the existence of an activation threshold within our VCSEL device and allow us to continue investigating any potential functionalities that may utilize this capability, such as input integration, as presented in the manuscript.


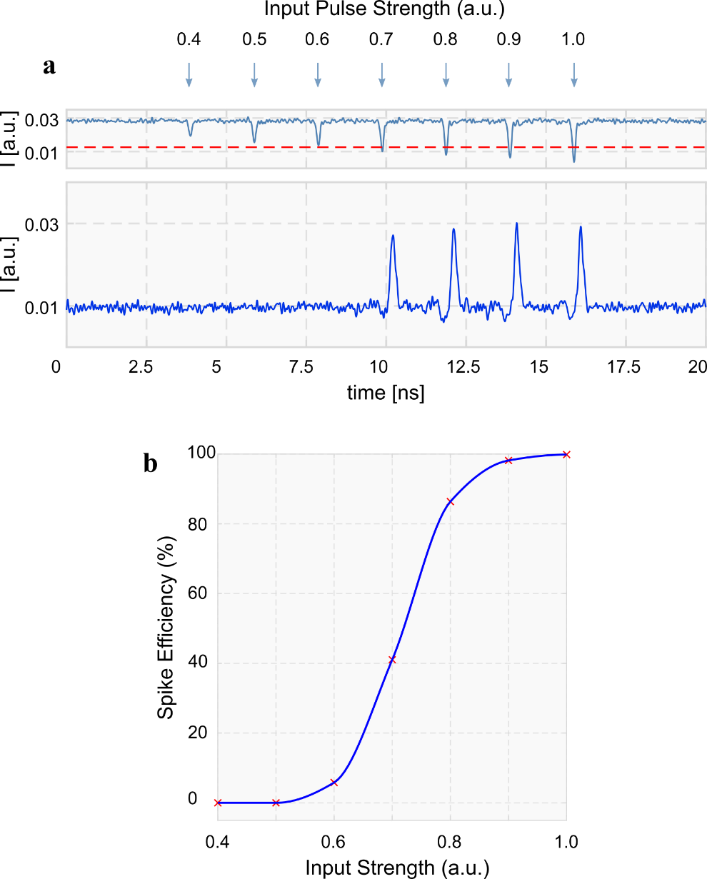


**Fig. S2 | Spike activation threshold in VCSEL-neurons.** **a** Captured time series showing the externally injected optical signal encoded with 7 (~100 ps) pulses of increasing intensity (top) and the corresponding VCSEL-neuron response (bottom). Red line is representative of the systems threshold for spike activation. **b** The efficiency curve of spike firing activations as a function of increasing input pulse intensity (stimulus strength).

**4. Coincidence Detection - Temporal Maps**

The manuscript demonstrates integration of temporally uncorrelated input pulse pairs for use in coincidence detection functionalities. In Fig. 3a of the main article file, an encoded input pattern comprised of two controls and 7 pairs of input pulses (with separations decreasing from ~840 ps to ~340 ps) was optically-injected into the VCSEL-neuron. Fig. 3a in the main article file plots the VCSEL-neuron’s input and output time traces for a single instance of encoded optical injection. Below in Fig. S3**,** we additionally provide the temporal map produced using all 172 consecutive cycles of the encoded optical injection, to better highlight the consistency and reproducibility of the VCSEL-neuron’s response.

Using a temporal folding parameter of 75 ns (the length of the generated input sequence), the map in Fig. S3 plots the continuous VCSEL-neuron’s output as a cycling pattern, where the intensity of the activated spikes is illustrated in colour (yellow representing high intensity peaks and blue representing stable output intensity). The constant yellow line observed at the top of the map indicates that a spiking response occurs consistently at the start of every cycle in response to the strong control pulse at the beginning of the encoded input pattern. The temporal map shows that very scarce spiking responses appear in response to input pulse pairs with large t_delay_ temporal separation. However, as the input pulse pair separation is decreased below ~490 ps (fifth input pulse pair), spiking events start to illicit more consistently. When the separation reaches ~340 ps (seventh input pulse pair), the system fires a fast spiking dynamic in response to the majority of the 172 consecutive input cycles. We have found that the spike occurrence rate can reach high rates in excess of 90% for the shortest pulses separations (~340 ps) which then drops as pulse separations increase towards larger values (~840 ps). With the fine tuning of experimental conditions we believe even higher spike occurrence rates could be reached by this experimental arrangement.

These results, similar to those in Fig. S2b, demonstrate the existence of an activation threshold and validate that integration, and therefore the overall contribution to spike activation, between temporally correlated inputs is stronger than temporally uncorrelated inputs with large separations (t_delay_). Additionally, as in the integration demonstration in Fig. 2 of the main manuscript, we would expect a reduced requirement for integration at experimental conditions closer to threshold. Hence, closer to threshold we would expect higher spike occurrence rates from the larger pulse separations.


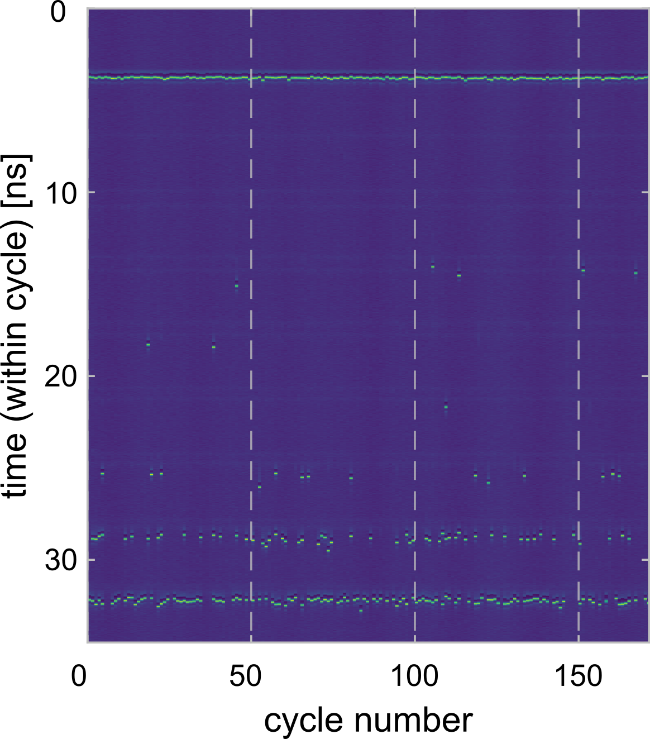


**Fig. S3 | Coincidence Detection Temporal Map.** A temporal map that plots a large continuous time traces as multiple cycling patterns. Spike intensity is represented using colour, with yellow indicating a spike crest and blue representing stable output level. Repeatable and consistent responses result in the formation of a line as the number of cycles increase. Data presented is consistent with that of Fig. 3a in the manuscript.

**5. Generation and Weighting of Input Data Patterns**

Input data sequences were generated using a column of values corresponding to the intensity of the desired signal in sample steps of 83.3 ps (12 GSa/s). The generated data sequences contained multiple 4-bit patterns separated by ~830 ps (10 zero samples), with all values falling within the DAC range of the AWG (-1 to 1). Each 4-bit pattern within the generated sequence was made up of 4 virtual inputs which were each assigned 2 samples. The first sample was set to the ‘true value’ of the input bit (1 or 0) and the second a return-to-zero value, to help distinguish bits in close proximity. The 4-bit patterns had a total duration of ~650 ps and were combined in a sequence with those of similar numbers of active ‘1’ bits. The 1, 2 and 3 active ‘1’ bit sequences contained 4, 6 and 4 patterns respectfully. The total length of each sequence measured ~30 ns (360 samples). The pattern sequences are grouped into their number of active bits to eliminate the requirement for multistep classification. Combining a mixture of 1, 2 and 3 active ‘1’ bit patterns would increase the complexity of the recognition task which then would require multiple recognition steps to eliminate false detections created by patterns containing similar active bits after weighting. In this first report we therefore demonstrate the recognition of patterns against those of similar active bits, using a single VCSEL neuron and a single classification step. The input data for all 1, 2 and 3 active ‘1’ bit sequences is shown encoded in optical injection below (Fig. S4a-c**)**.


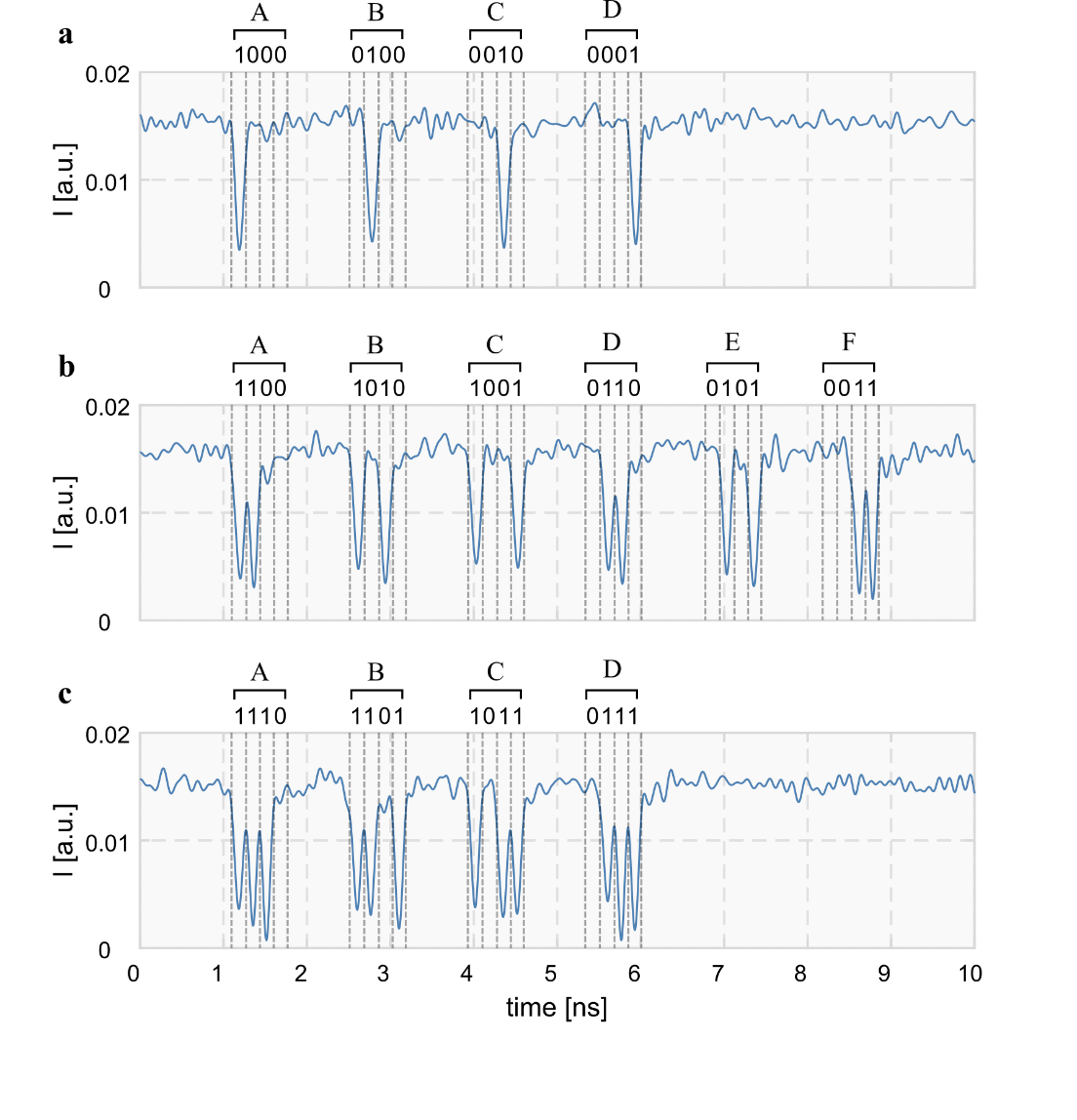


**Fig. S4 | Input Data Sequences.** Time series of input data sequences encoded within optical injection without weighting. Input data sequence of the **a** 1 active ‘1’ bit patterns, **b** 2 active ‘1’ bit patterns and **c** 3 active ‘1’ bit patterns. A total of 14 patterns are generated. Active ‘1’ bits are encoded as drops in optical injection intensity and ‘0’ bits as stable level injection intensity.

The weighting step is used to eliminate or reduce the contribution of incorrect bits to the firing and detection of a target pattern. To achieve this the input data sequences containing the 4-bit patterns were weighted using an array of weighting values. Each weighting value in the array corresponded to a sample point in the input data sequence (360 samples) and held a weighting value between -1 and 1. The weighting of individual bits within all 4-bit patterns was achieved using their corresponding array values, where W1, W2, W3 and W4 represent the weighting value of the first, second, third and fourth ‘true value’ bits respectfully. Weighting values W1–W4 were made consistent across all data patterns in a single input sequence or pattern grouping and all weighting values corresponding to non-patterns were set to zero. The product of the input data sequence and the weighting array was taken to produce the weighted data, prior to generation in the AWG. Weighting values were selected and adjusted through experimentation, hence the provided values may differ from ideal weight settings. The weighting value combination (W1-W4) used to detect each 4-bit pattern is unique as shown in the weight tables of Fig. S5a-c. Using unique weight combinations allows us to detect target patterns individually, eliminating all but the target pattern. More complex weighting schemes that allow for the recognition of groups of patterns may be possible, but are beyond the scope of the present work. Examples of weighted data for some 1, 2 and 3 active ‘1’ bit patterns are shown in the time series of Fig. S5d-f.


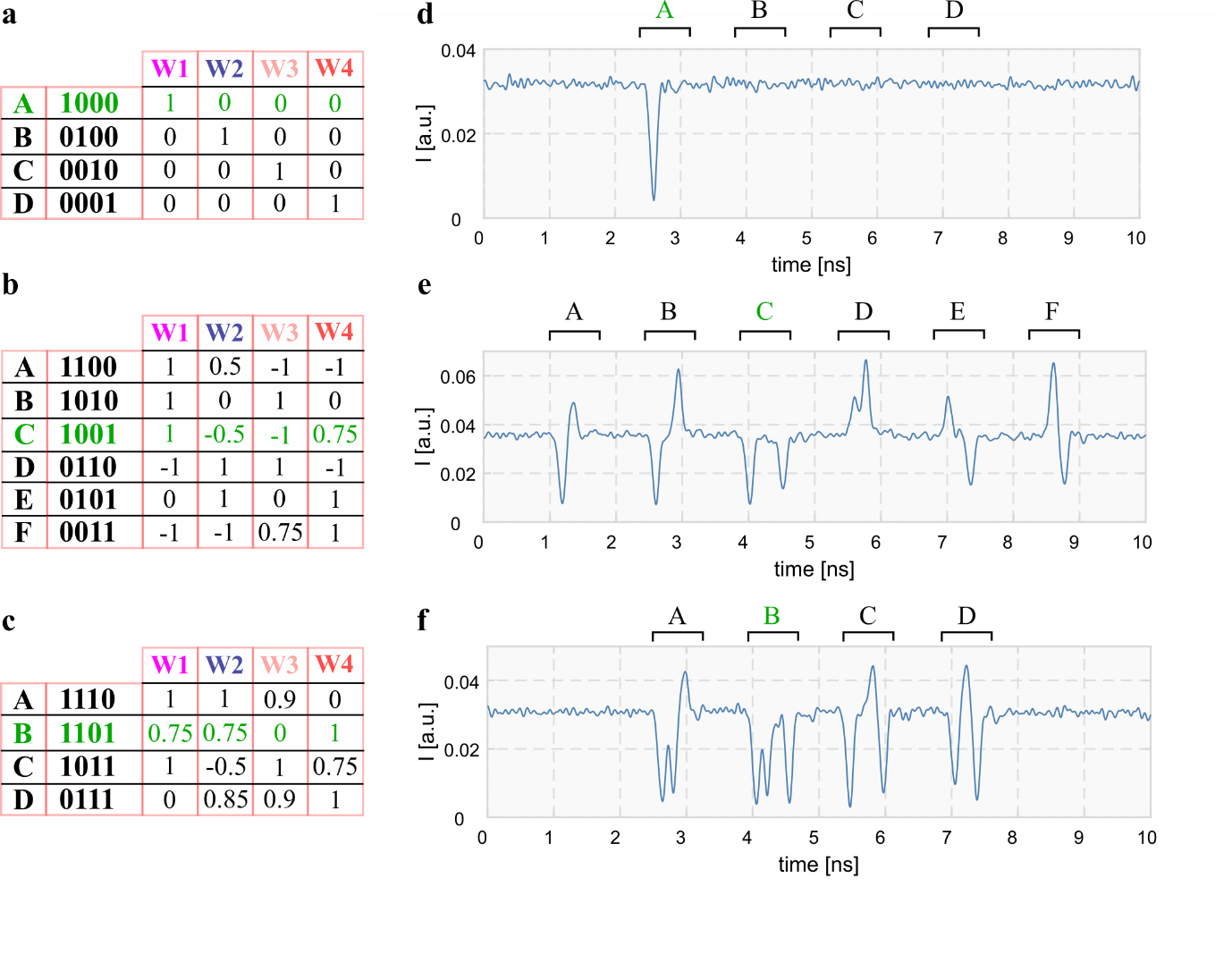


**Fig. S5 | Weight Tables and Weighted Input Data Patterns.** The weighting values used for the recognition of patterns of **a** 1, **b** 2 and **c** 3 active ‘1’ bits. **d-f** Time series of weighted input data encoded within optical injection. The target pattern of each plot and the associated weighting values are highlighted in green.

**6. Confusion Matrices**

The confusion matrices (as shown in Fig. 5 of the manuscript) represent the detection accuracy of each of the 4-bit input patterns. The horizontal axis represents different target patterns and a unique set of experimental weightings. In each case the weights were selected in such a way that only the target pattern (A to F on horizontal scale) should be detected by the VCSEL-neuron. The vertical axis represents the patterns detected by the system. Consequently each of the columns show the different patterns detected by the system for a single set of weights targeting a specific pattern. The values in the matrix represent the ratio of observed-to-expected pattern detections. In an ideal case, the confusion matrix would show '1.0' values on the diagonal and '0.0' values elsewhere, meaning the system returned neither any false negatives, nor any false positives. The sum of each column can be larger than unity as in each detection step the VCSEL-neuron can fire (detect) for multiple weighted input patterns.

**7. 4-bit Pattern Representation**

In the manuscript 4-bit data patterns are represented graphically by 2 x 2 grids of black and white pixels. Each pixel in the grid is representative of bit within the pattern. Black pixels represent active ‘1’ bits and white pixels represent ‘0’ bits. The pixel order is read by row with top left, top right, bottom left and bottom right representing the first, second, third and fourth bits respectfully. The representation of 4-bit pattern ‘1110’ can be found below in Fig. S6 below.


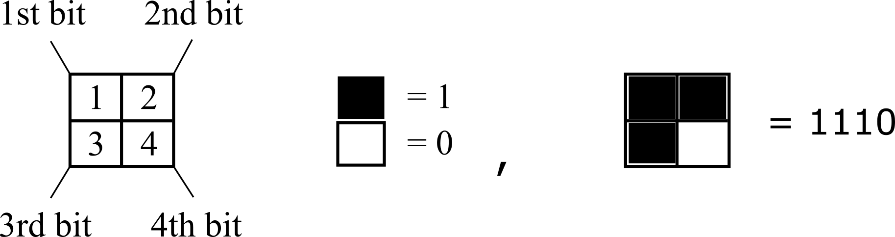


**Fig. S6 | 4-bit Pattern Representation.** Description of how 4-bit patterns are represented using 2x2 grids.
